# Association of isolated diastolic hypertension based on different guideline definitions with incident cardiovascular risk in a Chinese rural cohort

**DOI:** 10.1111/jch.14349

**Published:** 2021-12-15

**Authors:** Shiru Zhang, Sitong Liu, Yundi Jiao, Liqiang Zheng, Yingxian Sun, Zhaoqing Sun

**Affiliations:** ^1^ Department of Cardiology Shengjing Hospital of China Medical University Shenyang Liaoning Province P. R. China; ^2^ Department of Cardiology Department of Library and Department of Clinical Epidemiology Shengjing Hospital of China Medical University Shenyang Liaoning Province P. R. China; ^3^ Department of Cardiology The First Affiliated Hospital of China Medical University Shenyang Liaoning Province P. R. China

**Keywords:** blood pressure, cardiovascular disease, guidelines, isolated diastolic hypertension

## Abstract

The 2017 American College of Cardiology/American Heart Association (ACC/AHA) guideline lowered the threshold (systolic blood pressure [SBP] <130 mm Hg and diastolic blood pressure [DBP] ≥80 mm Hg) for isolated diastolic hypertension (IDH), whereas the 2018 Chinese guideline still recommends the old threshold (SBP <140 mm Hg and DBP ≥90 mm Hg). This study aimed to investigate the association between IDH, as defined by both guidelines, and the risk of incident cardiovascular disease (CVD) in rural areas of northeast China. This prospective study included participants whose baseline data were collected between 2004 and 2006. The exclusion criteria were baseline CVD, incomplete data, and systolic hypertension. The primary end point was incident CVD, a composite end point including nonfatal myocardial infarction (MI), nonfatal stroke, and CVD death. Multivariate Cox models were used to evaluate the association of IDH with CVD risk. The authors analyzed 19 688 participants (7140 participants with IDH) according to the ACC/AHA guideline. Compared with normotensive participants, individuals with ACC/AHA‐defined IDH were at a high risk of CVD (HR = 1.177, 95% CI: 1.035–1.339). A similar difference in CVD risk was noted when normotensive participants were compared with those with IDH, determined based on the 2018 Chinese guideline (HR = 1.218, 95% CI: 1.050–1.413). Similar results were found in participants who did not take antihypertensives at baseline. Moreover, IDH defined by either guideline was significantly associated with nonfatal MI. ACC/AHA‐defined IDH was associated with a risk of CVD, implying that blood pressure management should be improved in rural areas of China.

## 1 INTRODUCTION

Hypertension is a major risk factor for cardiovascular disease (CVD) and all‐cause death worldwide, and its prevalence and absolute burden are high.^1^, ^2^ At the same time, it is also a common preventable and easily controllable risk factor.^1^, ^2^, ^3^, ^4^ Isolated diastolic hypertension (IDH), a type of hypertension, is defined as only diastolic blood pressure (DBP) meets the criteria of hypertension.^5^, ^6^ Despite its low prevalence, IDH affects a non‐negligible number of Chinese people considering the large Chinese population; moreover, the awareness regarding IDH is low and it is often poorly managed.^7^ Previous studies have shown that IDH (SBP <140 mm Hg and DBP ≥90 mm Hg) is more prevalent in younger adults, and is associated with future systolic hypertension.^8^, ^9^, ^10^, ^11^, ^12^, ^13^ The 2017 hypertension guideline issued by the American College of Cardiology/American Heart Association (ACC/AHA) lowered the threshold of blood pressure and redefined IDH as SBP <130 mm Hg and DBP ≥80 mm Hg.^5^. This change has led to a two‐fold to four‐fold increase in the prevalence of IDH among adults in various countries.^8^, ^9^, ^14^

In contrast, the 2018 Chinese Guidelines for the Prevention and Treatment of Hypertension, (officially published in 2019) still recommended the original criteria (SBP <140 mm Hg and DBP ≥90 mm Hg) for the diagnosis of IDH.^6^ Although previous studies^15^, ^16^, ^17^ have confirmed the validity of lower thresholds in the 2017 ACC/AHA guideline, there are still inconsistent results as to whether IDH is associated with subsequent incident CVD.^8^, ^14^, ^18^, ^19^, ^20^, ^21^, ^22^ Previous studies by Wu and coworkers^9^ in Chinese adults, Kaneko and coworkers^18^ in Japanese adults and Lee and coworkers^19^ among young adults in South Korea have demonstrated IDH independently predicted adverse CVD events. However, McEvoy and coworkers^8^ and McGrath and coworkers^14^ examined the impact of IDH on CVD risk in 8703 American adults and 89 126 participants in the United Kingdom Biobank cohort, respectively, and found that IDH was not associated with subsequent incident CVD. Therefore, it is necessary to conduct more research to verify this association.

This study aimed to assess the association of IDH defined by different guidelines with the risk of incident CVD via an epidemiological study in rural areas of northeast China.

## 2 METHODS

### 2.1 Study population

**FIGURE 1:**
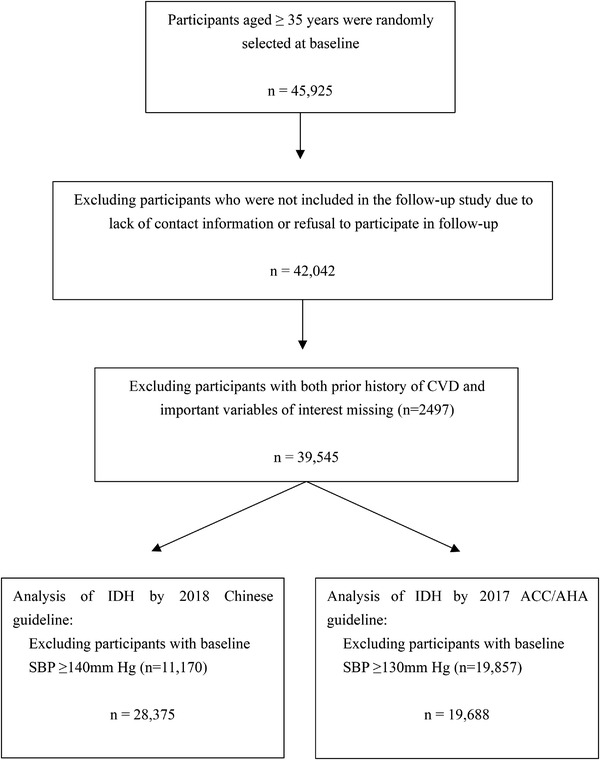
Flow chart of the exclusion process in Fuxin Country, China of participants analyzed

This was a large‐scale epidemiological prospective study that adopted a multistage, random, stratified clustering sampling scheme designed to determine the prevalence, incidence, and natural history of cardiovascular risk factors in eight towns and 84 rural villages of Fuxin County in Liaoning Province, China. The selection of the eight towns and 84 villages was as follows: towns were randomly selected from the northern, southern, western, eastern, and central regions of Fuxin. Thereafter, according to the population, three towns were selected from the southern region, two from the eastern region, and one from each of the other three regions. Next, 8–12 rural villages from each town were randomly selected from different geographical areas. For the longitudinal analyses in this study, we randomly enrolled 45 925 participants aged ≥35 years as a representative sample to collect baseline data from 2004 to 2006 and invited all investigators to attend the follow‐up study in 2008, 2010, and 2017.^23^, ^24^ We excluded participants without contact information or those who refused to participate in the follow‐up (*n* = 3883). Accordingly, there were 42 042(91.5%) participants who were followed up at least once. Of these, we further excluded those with a history of CVD at baseline, including coronary heart disease (CHD, ie, angina pectoris, myocardial infarction [MI], and arrhythmia) and stroke (ie, hemorrhagic stroke and ischemic stroke), omissions of important variables of interest and SBP within the range of hypertension (Figure 1).

This study was designed and conducted in accordance with the Helsinki Declaration, and the protocol was approved by the research ethics committee of China Medical University (2004PS51K). All participants or their proxies formally provided written informed consent.

### 2.2 Blood pressure measurement

After a 5‐min rest in the sitting position, three blood pressure measurements were taken for each participant by a trained and certified observer using an electric sphygmomanometer (HEM‐741C; OMRON, Tokyo, Japan); the average of the three measurements was considered for statistical analysis. Before these measurements, participants were directed to avoid drinking alcohol, coffee, or tea, and to refrain from smoking and exercise for at least 30 min.

### 2.3 Data collection and measurements

Trained researchers recorded and assessed data pertaining to the participants’ demographic variables (ie, age, sex, ethnicity, and education), lifestyle factors (ie, smoking status, alcohol use, and salt intake), medical history (ie, CHD, stroke, diabetes mellitus, and hyperlipidemia), and the use of antihypertensive medication, which was self‐reported information obtained via standardized questionnaires. The participants’ height and weight were measured when they wore light clothes and no shoes, and their body mass index (BMI) was calculated based on these measurements.^23^ We classified the level of education based on self‐reporting as primary school or below, middle school, and high school or above. Current smoking was defined as smoking at least one cigarette a day for at least 1 year. The criteria for the determination of drinking status have been described elsewhere.^25^ Salt is usually added to the diet as a condiment or used to make salted foods (as a preservative) in rural areas of China. By questioning the participants about the addition of salt to their daily diet and their consumption of salty foods, the researchers calculated the total amount of salt consumed by a family each year divided by the number of family members and obtained the estimated annual salt intake of the individual.^26^, ^27^ History of CHD or stroke was self‐reported and was confirmed by medical records. A history of diabetes mellitus was determined based on self‐report, a doctor's diagnosis, or the use of medication prescribed for diabetes. Information on antihypertensive medication use was self‐reported.

### 2.4 Study outcomes

The primary end point was incident CVD, which was a composite end point that included nonfatal MI, nonfatal stroke, and CVD death. During the follow‐up period, if more than one event occurred in a participant, the first event was considered the end point. In the specificity analysis of the end points, nonfatal MI, nonfatal stroke, CVD death, and all‐cause death were each considered secondary end points. In the secondary end point analysis, if a participant had different types of events, the first event of each type was considered the end point event. Details on the determination of death and MI and the classification of stroke in this study have been described elsewhere.^24^ Briefly, information about death was obtained by contacting the families directly and checking hospital records, which was based on the International Classification of Diseases, Ninth Revision, Clinical Modification (ICD‐9‐CM) code. We confirmed CVD death through autopsy reports, death certificates, medical records, or information provided by families. MI was defined based on the consensus document of the Joint European Society of Cardiology/American College of Cardiology Committee as a transient increase in laboratory markers specific to myocardial necrosis (CK‐MB or troponin T) in combination with ischemic symptoms and/or typical electrocardiographic signs (development of pathologic Q‐waves or ST segment elevation or depression). We used the WHO Multinational Monitoring of Trends and Determinants in CVD criteria to define stroke as rapidly developing signs of focal (or global) disturbance of cerebral function lasting >24 h (unless interrupted by surgery or death) with no apparent nonvascular cause. A nonfatal diagnosis was based on the records of events obtained from hospitalization data. All cardiovascular events were independently censored by the end point assessment committee, whose members were blinded to the baseline risk factor information of the study participants.

### 2.5 Statistical analysis

In this study, participants with IDH (according to each hypertension guideline) were compared with normotensive participants. We recorded the number of participants who met the IDH diagnostic criteria, based on both the 2017 ACC/AHA and the 2018 Chinese guidelines, and used both definitions to compare the demographic variables, lifestyle factors, medical history, and the use of antihypertensive medication of the participants who did and did not meet the IDH criteria. Normally distributed continuous variables are presented as the mean ± SD, and a *t*‐test was used for comparison between groups. A χ*^2^* test was used to assess categorical variables, which are presented as number (percentage). The cumulative incidence of CVD outcomes was determined based on the IDH category using Kaplan‐Meier curves.

Multivariate Cox proportional‐hazards models were used to describe and analyze the longitudinal associations between IDH, based on both guidelines, and the incidence of CVD, nonfatal MI, nonfatal stroke, CVD death, and all‐cause death outcomes; hazard ratios (HR) and 95% confidence intervals (95%CIs) were calculated. The proportional‐hazards assumption test based on Schoenfeld residuals was visually confirmed. Model 1 was adjusted according to age, sex, ethnicity, and educational level. Model 2 included all variables in model 1 plus current smoking, current alcohol use, current use of antihypertensive medication, baseline BMI, history of diabetes mellitus, history of hyperlipidemia, and salt intake. The adjusted variables of model 3 consisted of those in model 2 in addition to the SBP value at baseline.

We also conducted a series of subgroup analyses. First, the association of IDH, based on both definitions, with incident CVD outcomes was assessed after stratification of the participants according to baseline median age or sex. Second, we analyzed if IDH was associated with incident CVD for the subgroup that did not take antihypertensive drugs at baseline. All statistical analyses were performed using STATA (version 16, StataCorp LLC), and a two‐sided *p*‐value <.05 was considered statistically significant.

## 3 RESULTS

A total of 39 545 participants were enrolled after excluding participants with history of CVD and missing variables of interest (*n* = 2497) (Figure 1). We then excluded individuals with baseline SBP ≥140 mm Hg (*n* = 11 170) in order to analyze Chinese‐defined IDH. Thus, 28 375 participants with SBP <140 mm Hg (mean age 48.1 years, 14 409 [50.8%] male) were studied, and among them, 26 016 (91.7%) were normotensive and 2359 (8.3%) had IDH. Considering the 2017 ACC/AHA definition of IDH, after excluding participants with baseline SBP ≥130 mm Hg (*n* = 19 857), we analyzed 19 688 participants with SBP <130 mm Hg (mean age 47.2 years, 9,346 [47.5%] male), including 12,548 (63.7%) normotensive participants and 7140 (36.3%) participants with IDH. Overall, the prevalence of ACC/AHA‐defined IDH was 12.1% greater than the prevalence of IDH determined based on the Chinese definition.

Individuals with IDH as per either guideline were less likely to have a history of diabetes mellitus but were more likely to be male, Mongolian, current smokers, current drinkers, and have higher baseline SBP, DBP, and BMI (Table 1). When using the 2017 ACC/AHA definition, participants with IDH were more likely to have a higher salt intake, but participants with IDH as defined by the 2018 Chinese guideline tended to be older and more likely to have a history of hyperlipidemia (Table 1). Participants with ACC/AHA‐defined IDH were more likely than those with Chinese‐define IDH to be younger, more educated, have lower baseline SBP, DBP and BMI, but were less likely to be current drinkers or have a history of hyperlipidemia (Table 1).

**TABLE 1:** Baseline characteristics of participants based on IDH status by 2018 Chinese and 2017 ACC/AHA definitions

Characteristics	Normotension by Chinese definition	IDH by Chinese definition	*p* value^a^	Normotension by ACC/AHA definition	IDH by ACC/AHA definition	*p* value^b^	*p* value^c^
Participants, No. (%)	26016 (91.7)	2359 (8.3)		12548 (63.7)	7140 (36.3)		
Age, mean (SD), years	48.1±10.4	48.7±9.9	0.004	47.2±10.2	47.1±9.4	0.703	<0.001
Sex, No. (%)			0.001			<0.001	0.320
** **Male	13133 (50.5)	1276 (54.1)		5568 (44.4)	3778 (52.9)		
** **Female	12883 (49.5)	1083 (45.9)		6980 (55.6)	3362 (47.1)		
Ethnicity, No. (%)			0.019			<0.001	0.863
** **Han	0800 (80.0)	1832 (77.7)		10247 (81.7)	5583 (78.2)		
** **Mongolian	4897 (18.8)	500 (21.2)		2151 (17.1)	1477 (20.7)		
** **Others	319 (1.2)	27 (1.1)		150 (1.2)	80 (1.1)		
Education level, No. (%)			<0.001			0.043	<0.001
** **Primary school or below	10038 (38.6)	1006 (42.6)		4649 (37.0)	2564 (35.9)		
** **Middle school	14562 (56.0)	1214 (51.5)		7255 (57.8)	4157 (58.2)		
** **High school or above	1416 (5.4)	139 (5.9)		644 (5.1)	419 (5.9)		
Current smoking, No. (%)	10592 (40.7)	1046 (44.3)	0.001	4684 (37.3)	3009 (42.1)	<0.001	0.061
Current drinking, No. (%)	7992 (30.7)	857 (36.3)	<0.001	3362 (26.8)	2352 (32.9)	<0.001	0.003
Antihypertensive, No. (%)	116 (0.4)	70 (3.0)	<0.001	28 (0.2)	50 (0.7)	<0.001	<0.001
SBP, mean (SD), mm Hg	121.3±10.8	129.2±7.4	<0.001	114.8±9.5	120.4±6.3	<0.001	<0.001
DBP, mean (SD), mm Hg	76.1±7.5	93.2±4.7	<0.001	70.8±6.0	83.8±4.4	<0.001	<0.001
BMI, mean (SD), kg/m^2^	22.97±2.86	23.58±3.11	<0.001	22.71±2.81	23.24±2.89	<0.001	<0.001
Diabetes mellitus, No. (%)	72 (0.3)	8 (0.3)	0.584	34 (0.3)	14 (0.2)	0.306	0.210
History of hyperlipidemia, No. (%)	335 (1.3)	42 (1.8)	0.045	129 (1.0)	88 (1.2)	0.187	0.047
Salt intake, mean (SD), g/day	15.52±12.37	15.76±11.26	0.354	15.33±12.60	15.94±11.98	0.001	0.525

*Abbreviations*: ACC, American College of Cardiology; AHA, American Heart Association; BMI, body mass index; DBP, diastolic blood pressure; IDH, isolated diastolic hypertension; SBP, systolic blood pressure.

^a^ *p* value comparing those with normotension (SBP <140 mm Hg and DBP <90 mm Hg) to those with IDH (SBP <140 mm Hg and DBP ≥90 mm Hg), according to the definition of IDH by the 2018 Chinese Guidelines for Prevention and Treatment of Hypertension.

^b^ *p* value comparing those with normotension (SBP <130 mm Hg and DBP <80 mm Hg) to those with IDH (SBP <130 mm Hg and DBP ≥80 mm Hg), according to the definition of IDH by the 2017 ACC/AHA guideline.

^c^ *p* value comparing those with IDH by the 2018 Chinese definition vs those with IDH by the 2017 ACC/AHA definition.

**FIGURE 2:**
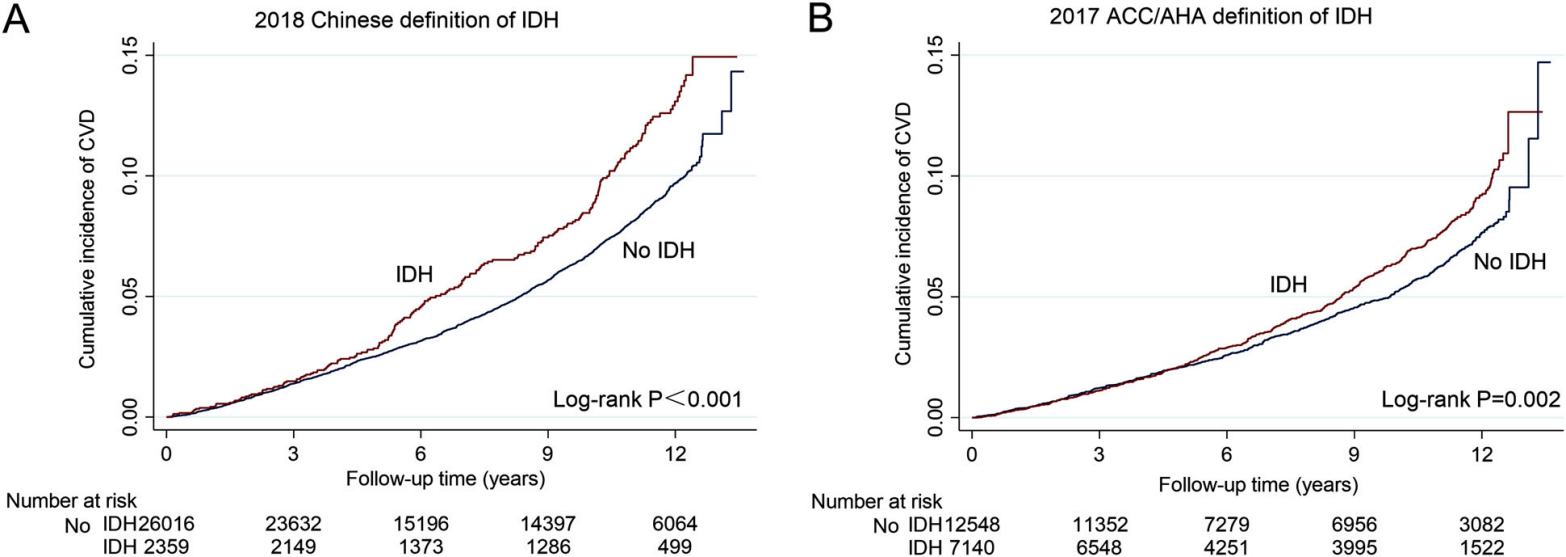
Cumulative incidence of CVD events based on both 2018 Chinese and 2017 ACC/AHA definitions of IDH (A.B)

During a median follow‐up of 11.3 years, a total of 1890 events were attributed to CVD among 28 375 participants with IDH that was diagnosed based on the 2018 Chinese guideline. There were 2059 all‐cause deaths, 95 nonfatal MIs, 962 nonfatal strokes, and 856 CVD deaths (Table S1). During a median follow‐up period of 11.3 years, a total of 1077 CVD events were recorded among 19 688 participants with IDH that was diagnosed based on the 2017 ACC/AHA recommendations; and among these, there were 1192 all‐cause deaths, 57 nonfatal MIs, 579 nonfatal strokes, and 453 CVD deaths (Table S1). The specific number and percentage of the primary and secondary end points under the two definitions are shown in Table S1. The Kaplan‐Meier cumulative incidence curves of the primary end point are provided in Figure 2. The cumulative incidence of CVD outcomes was higher in participants with IDH, regardless of the diagnosis criteria.

We assessed the associations between IDH and the different end point events and calculated HR (95%CI) using the Cox models (Table 2). As shown in Table 2 and Figure S1, compared with normotension, the risks of incident CVD (HR = 1.177, 95% CI: 1.035–1.339) and incident nonfatal MI (HR = 2.466, 95% CI: 1.389–4.379) increased significantly in the ACC/AHA‐defined IDH group in model 3, but IDH was not associated with nonfatal stroke (HR = 1.140, 95% CI: 0.957–1.358), CVD death (HR = 1.182, 95% CI: 0.968–1.443), or all‐cause death (HR = 1.029, 95% CI: 0.908–1.165]). Compared with normotension as defined by the 2018 Chinese guideline, IDH was associated with significantly increased risks of incident CVD (HR = 1.218, 95% CI: 1.050–1.413) and incident nonfatal MI (HR = 1.808, 95% CI: 1.005–3.25), but there were no significant increases for incident nonfatal stroke (HR = 1.214, 95% CI: 0.988–1.493), CVD death (HR = 1.174, 95% CI: 0.935–1.473) or all‐cause death (HR = 1.112, 95% CI: 0.956–1.292) in model 3 (Table 2).

**TABLE 2:** Associations between IDH, base on 2018 chinese and 2017 ACC/AHA definitions, and incident end point events^c^

		CVD	Nonfatal MI	Nonfatal stroke	CVD death	All‐cause death
Definition of IDH	Adjustment model	Hazard ratio (*95%CI*)				
IDH by Chinese definition	Model 1^a^	1.343 (1.162–1.552)	2.076 (1.195–3.605)	1.385 (1.133–1.692)	1.252 (1.002–1.565)	1.150 (0.992–1.333)
	Model 2^b^	1.305 (1.128–1.510)	1.781 (1.007–3.151)	1.308 (1.068–1.601)	1.257 (1.005–1.572)	1.155 (0.996–1.339)
	Model 3^c^	1.218 (1.050–1.413)	1.808 (1.005–3.251)	1.214 (0.988–1.493)	1.174 (0.935–1.473)	1.112 (0.956–1.292)
IDH by ACC/AHA definition	Model 1^a^	1.226 (1.085–1.386)	2.476 (1.459–4.200)	1.220 (1.033–1.441)	1.202 (0.993–1.455)	1.016 (0.902–1.145)
	Model 2^b^	1.202 (1.062–1.359)	2.206 (1.291–3.771)	1.180 (0.998–1.396)	1.205 (0.994–1.460)	1.038 (0.920–1.170)
	Model 3^c^	1.177 (1.035–1.339)	2.466 (1.389–4.379)	1.140 (0.957–1.358)	1.182 (0.968–1.443)	1.029 (0.908–1.165)

*Abbreviations*: ACC, American College of Cardiology; AHA, American Heart Association; CVD, cardiovascular disease; DBP, diastolic blood pressure; IDH, isolated diastolic hypertension; MI, myocardial infarction; SBP, systolic blood pressure.

^a^ Model 1 is adjusted for age, sex, ethnicity and educational level.

^b^ Model 2 is adjusted for model 1 plus current smoking status, current alcohol consumption status, antihypertensive medication use status, baseline body mass index, diabetes mellitus status, history of hyperlipidemia and salt intake.

^c^ Model 3 is adjusted for model 2 plus baseline SBP value.

^c^All comparisons are to participants in the rural cohort in Northeast China with normotension (when studying IDH based on the 2018 Chinese Guidelines for Prevention and Treatment of Hypertension, this group consists of those with SBP <140 mm Hg and DBP <90 mm Hg; when studying IDH by the 2017 ACC/AHA definition, this group consists of those with SBP <130 mm Hg and DBP <80 mm Hg).

**FIGURE 3:**
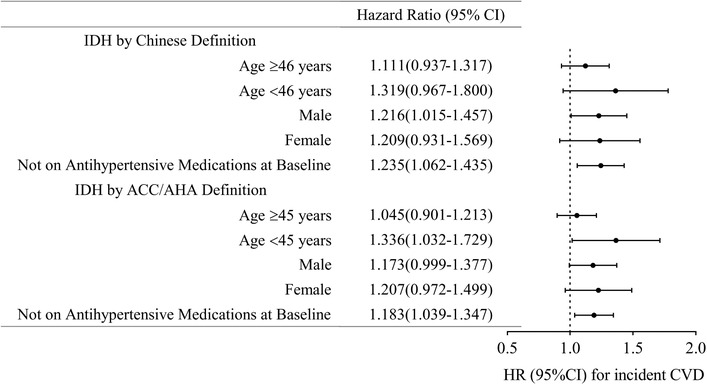
Subgroup analyses of hazard ratio (95%CI) of the association of IDH, by both 2018 Chinese and 2017 ACC/AHA definitions, with incident CVD according to median age, sex, or not being on baseline antihypertensive medications*

A subgroup analysis of the associations between IDH and the risk of end point events was also performed for participants who were not on antihypertensive medication at baseline, and similar findings were noted (Table S2). IDH, defined by both guidelines, was significantly associated with incident CVD and incident nonfatal MI. Further, IDH, defined by the 2018 Chinese guideline, was associated with a higher risk of incident nonfatal stroke (HR = 1.249, 95% CI: 1.014–1.538) in model 3 (Table S2). We also assessed the association between IDH, defined by both guidelines, and incident CVD in subgroups stratified by median age at baseline and sex (Figure 3). We observed that IDH diagnosed as the 2018 Chinese definition was not associated with CVD stratified based on median age; however, in the male population, it was significantly associated with incident CVD (HR = 1.216, 95% CI: 1.015–1.457) (Figure 3). In addition, there were different results in the ACC/AHA‐defined IDH group, where IDH was significantly associated with a higher risk of incident CVD only in those aged <45 years (HR = 1.336, 95% CI: 1.032–1.729) (Figure 3). The findings from the Cox proportional‐hazards models are presented in Tables S2–S4.

## 4 DISCUSSION

In this study, we assessed the associations between IDH defined by two guidelines and the risk of incident CVD events in rural areas of northeast China. The results showed that the prevalence of IDH was significantly higher with the 2017 ACC/AHA definition than with the 2018 Chinese definition. IDH, based on both definitions, showed significant associations with incident CVD and incident nonfatal MI. In the subgroup analysis, we observed similar results in participants who did not take antihypertensive therapies at baseline, and the CVD risk was most notable among men when considering the 2018 Chinese definition of IDH and among adults aged <45 years when considering the 2017 ACC/AHA guideline. The results of this study provide supplementary evidence to confirm the association between IDH and increased CVD risk.

A previous study^21^ indicated that although elevated SBP had a greater impact on the prognosis of patients, systolic or diastolic hypertension independently increased the CVD risk in adults. At present, there are conflicting results from previous large epidemiological studies on whether the 2017 ACC/AHA guideline‐based IDH is associated with an increased risk of CVD outcomes.^8^, ^9^, ^14^, ^18^, ^19^ The studies by McEvoy and coworkers^8^ in American adults and McGrath and coworkers^14^ in the United Kingdom Biobank cohort showed that there was no association between the 2017 ACC/AHA guideline‐defined IDH and an increased risk of CVD outcomes. This conclusions was contrary to our findings, and these differences with our findings might be attributable to the differences in the study population, study design, end points, follow‐up time, and location.

In addition, considering pathophysiology, with increased age, SBP gradually increases, while DBP gradually decreases.^28^ The results of a well‐known study of the Framingham population^29^ showed that the association between DBP and CVD risk was affected by age. In populations <50 years old, DBP could predict the risk of CHD. This result was also confirmed in our subgroup study. However, in the study by McEvoy and coworkers^8^, the youngest participant in the study population was 48 years old, and the average age of the IDH population was >50 years. Thus, this age range did not accurately represent the age group with a high incidence of IDH, which might also be one reason why IDH was not associated with CVD outcomes in their study.

Contrary to these studies that reported an absence of any association between IDH and CVD outcomes, the following studies showed a significant association between IDH and CVD outcomes. A large‐scale, epidemiological study in Japan^18^ showed that IDH as defined by the 2017 ACC/AHA guideline was associated with an increased risk of CVD events. This conclusions is similar to our study results, but the primary end points in our study and theirs were different. Moreover, in their study^18^, incident CVD was defined as MI, angina pectoris, and stroke. In contrast, we defined incident CVD as a composite end point that included nonfatal MI, nonfatal stroke, and CVD death. In the adult Chinese community, Wu and coworkers^9^ found a significant association between IDH (diagnosed based on 2017 ACC/AHA) and incident cerebral hemorrhage and total incident CVD, whereas our study focused on rural northeast China and found that IDH was significantly associated with incident CVD and incident nonfatal MI. All these findings, considered together, suggest that IDH is markedly associated with a higher risk of incident CVD and thus highlight the importance of improving the management of blood pressure.

This Chinese rural cohort study has several limitations. First, the participants come from the rural areas of northeast China, so the findings cannot be generalized to other populations without verification; our findings must be validated by assessing this association in populations as well. Second, the population of this study was restricted to adults aged ≥35 years at baseline, which makes it difficult to apply our findings to younger adults. A previous study has confirmed that IDH is associated with the risk of incident CVD among young adults.^19^ Third, we lacked information on some potential confounders such as blood biochemical data at baseline; further research is needed in this regard. Finally, the focus of this study was IDH, and the relative merits of the SBP cutoff value recommended in the guidelines were not evaluated.

## 5 CONCLUSIONS

In a rural population in northeast China, the prevalence of IDH was higher when diagnosed using the 2017 ACC/AHA guideline than when using the 2018 Chinese guideline. Individuals with IDH regardless of the diagnosis criteria had a higher risk of CVD events.

## CONFLICT OF INTEREST

The authors declare no conflicts of interest

## AUTHOR CONTRIBUTIONS

Shiru Zhang wrote the first draft of the manuscript. Zhaoqing Sun, Yingxian Sun conceived and designed the study. Sitong Liu, Yundi Jiao, Liqiang Zheng supervised the analysis and contributed to the discussion. All authors have contributed to the study according to international consensus on authorship, approved the version to be published, and agreed to be accountable for all aspects of the work in ensuring that questions related to the accuracy or integrity of any part of the work are appropriately investigated and resolved.

## Supporting information

Supplementary information
