# Supplementary material for: Association of isolated diastolic hypertension based on different guideline definitions with incident cardiovascular risk in a Chinese rural cohort
Source: J Clin Hypertens (Greenwich). 2021 Dec 15;24(1):18–25. doi: 10.1111/jch.14349 (PMC8783363; doi:10.1111/jch.14349)
Supplement: Supplementary file 1 — Supplementary information [file JCH-24-18-s001.docx]

Supporting Information for online publication only

Methods.

Table S1. Cardiovascular Disease Events

Table S2. Associations of IDH, by 2018 Chinese and 2017 ACC/AHA Definitions, With Incident Endpoint Events, according to participants who not on Antihypertensive Medications at Baseline

Table S3. Associations Between IDH, by 2018 Chinese and 2017 ACC/AHA Definitions, and Incident CVD Events, After Stratification by Median Age

Table S4. Associations Between IDH, by 2018 Chinese and 2017 ACC/AHA Definitions, and Incident CVD Events, After Stratification by Sex

Figure S1. Graphical Abstract Image

Table S1. Cardiovascular Disease Events*

|  | Normotension by Chinese Definition | IDH by Chinese Definition | *p* value^a^ | Normotension by ACC/AHA Definition | IDH by ACC/AHA Definition | *p* value^b^ |
| --- | --- | --- | --- | --- | --- | --- |
| CVD | 1684(6.5) | 206(8.7) | <0.001 | 640(5.1) | 437(6.1) | 0.002 |
| Nonfatal MI | 80(0.3) | 15(0.6) | 0.008 | 24(0.2) | 33(0.5) | 0.001 |
| Nonfatal Stroke | 854(3.3) | 108(4.6) | 0.001 | 341(2.7) | 238(3.3) | 0.014 |
| CVD Death | 770(3.0) | 86(3.6) | 0.062 | 278(2.2) | 175(2.5) | 0.289 |
| All-cause Death | 1864(7.2) | 195(8.3) | 0.048 | 765(6.1) | 427(6.0) | 0.742 |

Abbreviations: IDH = isolated diastolic hypertension, ACC = American College of Cardiology, AHA = American Heart Association, CVD = cardiovascular disease, MI = myocardial infarction, SBP = systolic blood pressure, DBP = diastolic blood pressure.

*Values are n (%).

^a^*p* value comparing those with normotension (SBP <140 mm Hg and DBP <90 mm Hg) to those with IDH (SBP <140 mm Hg and DBP ≥90 mm Hg), according to the definition of IDH by the 2018 Chinese Guidelines for Prevention and Treatment of Hypertension.

^b^*p* value comparing those with normotension (SBP <130 mm Hg and DBP <80 mm Hg) to those with IDH (SBP <130 mm Hg and DBP ≥80 mm Hg), according to the definition of IDH by the 2017 ACC/AHA guideline.

Table S2. Associations of IDH, by 2018 Chinese and 2017 ACC/AHA Definitions, With Incident Endpoint Events, according to participants who not on Antihypertensive Medications at Baseline *

| Definition of IDH | Adjustment Model | CVD | Nonfatal MI | Nonfatal Stroke | CVD Death | All-cause Death |
| --- | --- | --- | --- | --- | --- | --- |
|  |  | Hazard Ratio(95%CI) | | | | |
| IDH by Chinese Definition | Model 1^a^ | 1.337(1.154-1.550) | 1.906(1.059-3.431) | 1.386(1.131-1.700) | 1.261(1.006-1.579) | 1.149(0.989-1.335) |
|  | Model 2^b^ | 1.326(1.144-1.537) | 1.887(1.047-3.399) | 1.350(1.100-1.655) | 1.266(1.011-1.587) | 1.157(0.996-1.344) |
|  | Model 3^c^ | 1.235(1.062-1.435) | 1.885(1.029-3.456) | 1.249(1.014-1.538) | 1.182(0.940-1.486) | 1.115(0.958-1.299) |
| IDH by ACC/AHA Definition | Model 1^a^ | 1.216(1.075-1.376) | 2.307(1.336-3.983) | 1.224(1.035-1.447) | 1.194(0.986-1.447) | 1.016(0.901-1.145) |
|  | Model 2^b^ | 1.206(1.066-1.366) | 2.222(1.285-3.843) | 1.199(1.013-1.419) | 1.202(0.991-1.457) | 1.037(0.919-1.169) |
|  | Model 3^c^ | 1.183(1.039-1.347) | 2.489(1.383-4.477) | 1.158(0.971-1.387) | 1.181(0.966-1.444) | 1.028(0.907-1.165) |

Abbreviations: IDH = isolated diastolic hypertension, ACC = American College of Cardiology, AHA = American Heart Association, CVD = cardiovascular disease, MI = myocardial infarction, SBP = systolic blood pressure, DBP = diastolic blood pressure.

*All comparisons are to participants in the rural cohort in Northeast China with normotension (when studying IDH based on the 2018 Chinese Guidelines for Prevention and Treatment of Hypertension, this group consists of those with SBP <140 mm Hg and DBP <90 mm Hg; when studying IDH by the 2017 ACC/AHA definition, this group consists of those with SBP <130 mm Hg and DBP <80 mm Hg).

^a^Model 1 is adjusted for age, sex, ethnicity and educational attainment.

^b^Model 2 is adjusted for model 1 plus current smoking status, current alcohol consumption status, baseline body mass index, diabetes mellitus status, history of hyperlipidemia and salt intake.

^c^Model 3 is adjusted for model 2 plus baseline SBP value.

Table S3. Associations Between IDH, by 2018 Chinese and 2017 ACC/AHA Definitions, and Incident CVD Events, After Stratification by Median Age*

| Stratification by Median Age | Definition of IDH | Adjustment Model | Hazard Ratio(95%CI) |
| --- | --- | --- | --- |
| Age less than median | IDH by Chinese Definition | Model 1^a^ | 1.597(1.189-2.146) |
|  |  | Model 2^b^ | 1.475(1.088-1.998) |
|  |  | Model 3^c^ | 1.319(0.967-1.800) |
|  | IDH by ACC/AHA Definition | Model 1^a^ | 1.415(1.109-1.806) |
|  |  | Model 2^b^ | 1.377(1.077-1.761) |
|  |  | Model 3^c^ | 1.336(1.032-1.729) |
| Age greater than or equal to median | IDH by Chinese Definition | Model 1^a^ | 1.225(1.038-1.447) |
|  |  | Model 2^b^ | 1.217(1.029-1.438) |
|  |  | Model 3^c^ | 1.111(0.937-1.317) |
|  | IDH by ACC/AHA Definition | Model 1^a^ | 1.074(0.933-1.237) |
|  |  | Model 2^b^ | 1.068(0.926-1.231) |
|  |  | Model 3^c^ | 1.045(0.901-1.213) |

Abbreviations: IDH = isolated diastolic hypertension, ACC = American College of Cardiology, AHA = American Heart Association, CVD = cardiovascular disease, SBP = systolic blood pressure, DBP = diastolic blood pressure.

*All comparisons are to participants in the rural cohort in Northeast China with normotension (when studying IDH based on the 2018 Chinese Guidelines for Prevention and Treatment of Hypertension, this group consists of those with SBP <140 mm Hg and DBP <90 mm Hg; when studying IDH by the 2017 ACC/AHA definition, this group consists of those with SBP <130 mm Hg and DBP <80 mm Hg).

^a^Model 1 is adjusted for sex, ethnicity and educational attainment.

^b^Model 2 is adjusted for model 1 plus current smoking status, current alcohol consumption status, antihypertensive medication use status, baseline body mass index, diabetes mellitus status, history of hyperlipidemia and salt intake.

^c^Model 3 is adjusted for model 2 plus baseline SBP value.

Table S4. Associations Between IDH, by 2018 Chinese and 2017 ACC/AHA Definitions, and Incident CVD Events, After Stratification by Sex*

| Stratification by Sex | Definition of IDH | Adjustment Model | Hazard Ratio(95%CI) |
| --- | --- | --- | --- |
| Male | IDH by Chinese Definition | Model 1^a^ | 1.325(1.111-1.581) |
|  |  | Model 2^b^ | 1.304(1.091-1.557) |
|  |  | Model 3^c^ | 1.216(1.015-1.457) |
|  | IDH by ACC/AHA Definition | Model 1^a^ | 1.199(1.029-1.397) |
|  |  | Model 2^b^ | 1.195(1.025-1.394) |
|  |  | Model 3^c^ | 1.173(0.999-1.377) |
| Female | IDH by Chinese Definition | Model 1^a^ | 1.380(1.072-1.777) |
|  |  | Model 2^b^ | 1.292(1.000-1.668) |
|  |  | Model 3^c^ | 1.209(0.931-1.569) |
|  | IDH by ACC/AHA Definition | Model 1^a^ | 1.276(1.040-1.565) |
|  |  | Model 2^b^ | 1.229(1.000-1.511) |
|  |  | Model 3^c^ | 1.207(0.972-1.499) |

Abbreviations: IDH = isolated diastolic hypertension, ACC = American College of Cardiology, AHA = American Heart Association, CVD = cardiovascular disease, SBP = systolic blood pressure, DBP = diastolic blood pressure.

*All comparisons are to participants in the rural cohort in Northeast China with normotension (when studying IDH based on the 2018 Chinese Guidelines for Prevention and Treatment of Hypertension, this group consists of those with SBP <140 mm Hg and DBP <90 mm Hg; when studying IDH by the 2017 ACC/AHA definition, this group consists of those with SBP <130 mm Hg and DBP <80 mm Hg).

^a^Model 1 is adjusted for age, ethnicity and educational attainment.

^b^Model 2 is adjusted for model 1 plus current smoking status, current alcohol consumption status, antihypertensive medication use status, baseline body mass index, diabetes mellitus status, history of hyperlipidemia and salt intake.

^c^Model 3 is adjusted for model 2 plus baseline SBP value.


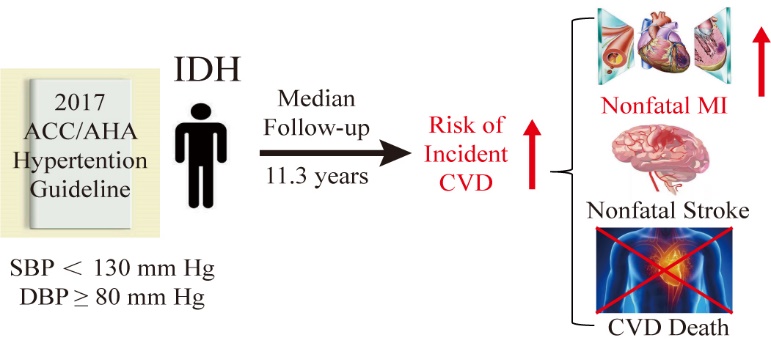


Figure S1. Graphical Abstract Image
